# First report of genetic diversity and risk factor analysis of equine piroplasm infection in equids in Jilin, China

**DOI:** 10.1186/s13071-020-04338-1

**Published:** 2020-09-09

**Authors:** Shaowei Zhao, Hao Wang, Shuang Zhang, Suzhu Xie, Hang Li, Xuancheng Zhang, Lijun Jia

**Affiliations:** Laboratory of Veterinary Microbiology, Department of Veterinary Medicine, Agriculture College of Yanbian University, No. 977 Park Road, Yanji, 133000 China

**Keywords:** Equine piroplasmosis, *Theileria equi*, Risk factors, Genetic diversity, Epidemiology

## Abstract

**Background:**

Equine piroplasmosis (EP) is a tick-borne hemoprotozoan disease of equids, caused by *Theileria equi* and *Babesia caballi*. Equine piroplasmosis represents a serious challenge to the equine industry due to important economic losses worldwide. The present study aimed to evaluate the prevalence of *Theileria equi* and *Babesia caballi* infections in equids from Jilin Province, China.

**Methods:**

A total of 220 blood samples (192 horses and 28 donkeys/mules) were collected from March 2018 to October 2019 in five districts of Jilin Province and analyzed by PCR. Potential risk factors, including the region, sex, management, and host species of the animals were assessed in relation to *T. equi* infection. Moreover, the V4 hypervariable region of the *T. equi 18S* rRNA gene was analyzed to identify specific genotypes.

**Results:**

The overall prevalence of *T. equi* in equids was 27.7%, whereas *B. caballi* infection was not identified. The district with the highest positive rate was Baicheng (43.3%), followed by Tonghua (28.9%), Yanbian (26.4%), Jilin (23.3%), and Liaoyuan (20.9%). The sex of the animals and farm management were identified as main risk factors, which were significantly associated with the prevalence of Equine piroplasmosis (*P* < 0.05). The risk factor analysis indicated that the females were at a higher risk (OR: 2.48, 95% CI: 1.17–5.27) of being infected compared to the males, whereas the organized farm was protective factor (OR: 0.42, 95% CI: 0.22–0.80). The phylogenetic analyses revealed that there were two *T. equi* genotypes (A and E) in Jilin.

**Conclusions:**

Our findings provided important epidemiological data for the prevention and control of *T. equi* infection in Jilin, China.
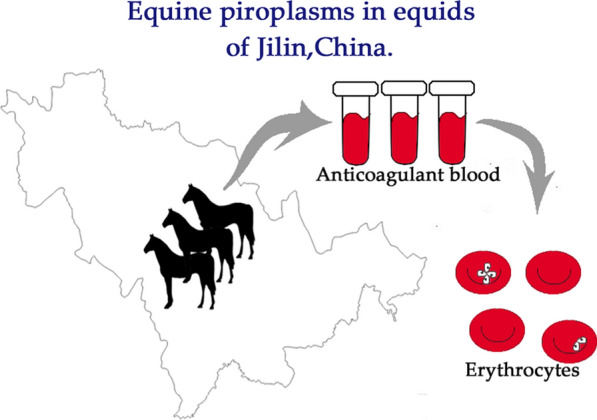

## Background

Equine piroplasmosis (EP) is a tick-borne disease caused by the hemoprotozoan parasites, *Theileria equi* and *Babesia caballi*, which causes fever, hemolytic anemia, jaundice, inappetence, and hemoglobinuria in all equid species (horses, donkeys, mules and zebras) [1]. Both hemoparasites are obligate intraerythrocytic protozoans classified within the phylum Apicomplexa, and are primarily transmitted by ixodid ticks belonging to the genera *Dermacentor*, *Hyalomma* and *Rhipicephalus* [2]. Equids infected with *T. equi* may represent long-term reservoirs, even if they have recovered from an acute or early infection. However, *B. caballi* causes a self-limiting infection that is naturally cleared by the horse’s immune system after 12 to 42 months post-infection [3]. Moreover, horses infected with equine piroplasmosis are strictly restricted for trade and sport. The causal agents of EP have been reported for more than 100 years, and this disease is widely distributed throughout Central and South America, Africa, Asia, Middle East and southern Europe, where it causes a substantial economic impact to the equine industry [4].

According to the Food and Agriculture Organization (FAO) statistics, the breeding stock of donkeys and horses in China was ranked second and third in the world, respectively [5]. The horse industry is primarily distributed in areas of northeast, north, and northwest China. Recently, the horse industry has displayed a wide range of development, which is not limited to agricultural production, but also the tourism, entertainment, competition, and food industries. Equine piroplasmosis is considered to be a notifiable disease by the World Organization for Animal Health (OIE), which has been reported in many regions of China, with prevalence rates varying from 10.9–80.1% (Table 1). The prevalence of EP was significantly higher in regions, such as Ningxia 80.1% [6], Guizhou 74.0% [7], and Yunnan 73.8% [6]. In addition, Xinjiang, Shanxi, and Hebei were also considered endemic areas of EP, with a prevalence of 40.8% [8], 44.3% and 54.7% [6], respectively.

**Table 1:** Prevalence of equine piroplasmosis in each area of China

Region	Diagnostic method	Positive rates (%)			References
		*T. equi* (95% CI)	*B. caballi* (95% CI)	Total (95% CI)	
Xinjiang	PCR	40.8 (37.2–44.4)	na	40.8 (37.2–44.4)	[8]
Yunnan	ELISA	37.6 (32.1–43.1)	67.1 (61.7–72.5)	73.8 (68.8–78.8)	[6]
Guangdong	ELISA	7.7 (6.2–9.2)	3.2 (2.2–4.2)	10.9 (9.1–12.7)	[11]
Shanghai	ELISA	29.2 (23.2–35.3)	na	29.2 (23.2–35.3)	[12]
Shanxi	ELISA	1.0 (0.0–2.5)	44.3 (37.2–51.4)	44.3 (37.2–51.4)	[6]
Hebei	ELISA	30.2 (17.4–43.0)	28.3 (15.8–40.8)	54.7 (40.9–68.6)	[6]
Jiangsu	ELISA	4.0 (1.4–6.5)	35.2 (29.0–41.5)	37.4 (31.1–43.8)	[6]
Beijing	ELISA	2.0 (0.0–4.3)	34.2 (26.5–41.9)	34.9 (27.2–42.6)	[6]
Guizhou	PCR	74.0 (66.1–81.8)	11.4 (5.7–17.1)	74.0 (66.1–81.8)	[7]
Ningxia	ELISA	4.0 (1.3–6.7)	77.6 (71.8–83.4)	80.1 (74.5–85.7)	[6]
Gansu	PCR	28.1 (22.4–33.8)	2.9 (0.8–5.0)	31.0 (25.1–36.9)	[13]

*Abbreviation*: na, not available

There are currently several diagnostic methods that can be used to diagnose infection with equine piroplasm, including thin smear examination, complement fixation test (CFT), immunofluorescent antibody test (IFAT), enzyme linked immunosorbent assay (ELISA), and polymerase chain reaction (PCR). The application of PCR assays, which target equine piroplasm genes, such as the *EMA-1* gene, *BC-48* gene, and *18S* ribosomal RNA (rRNA), have demonstrated higher analytical sensitivity and specificity than serological and microscopic detection [9, 10]. Although EP is an endemic disease among equids in Jilin Province, there are few studies involving the molecular epidemiology and interspecific genetic diversity of equine piroplasm. Thus, the aim of the present study was to determine the prevalence of equine piroplasm in equids from Jilin province in the northeastern region of China. We also sought to determine the risk factors associated with the occurrence of infection and genotypes of equine piroplasm in this region.

## Methods

### Study area and sample collection

**Fig. 1:**
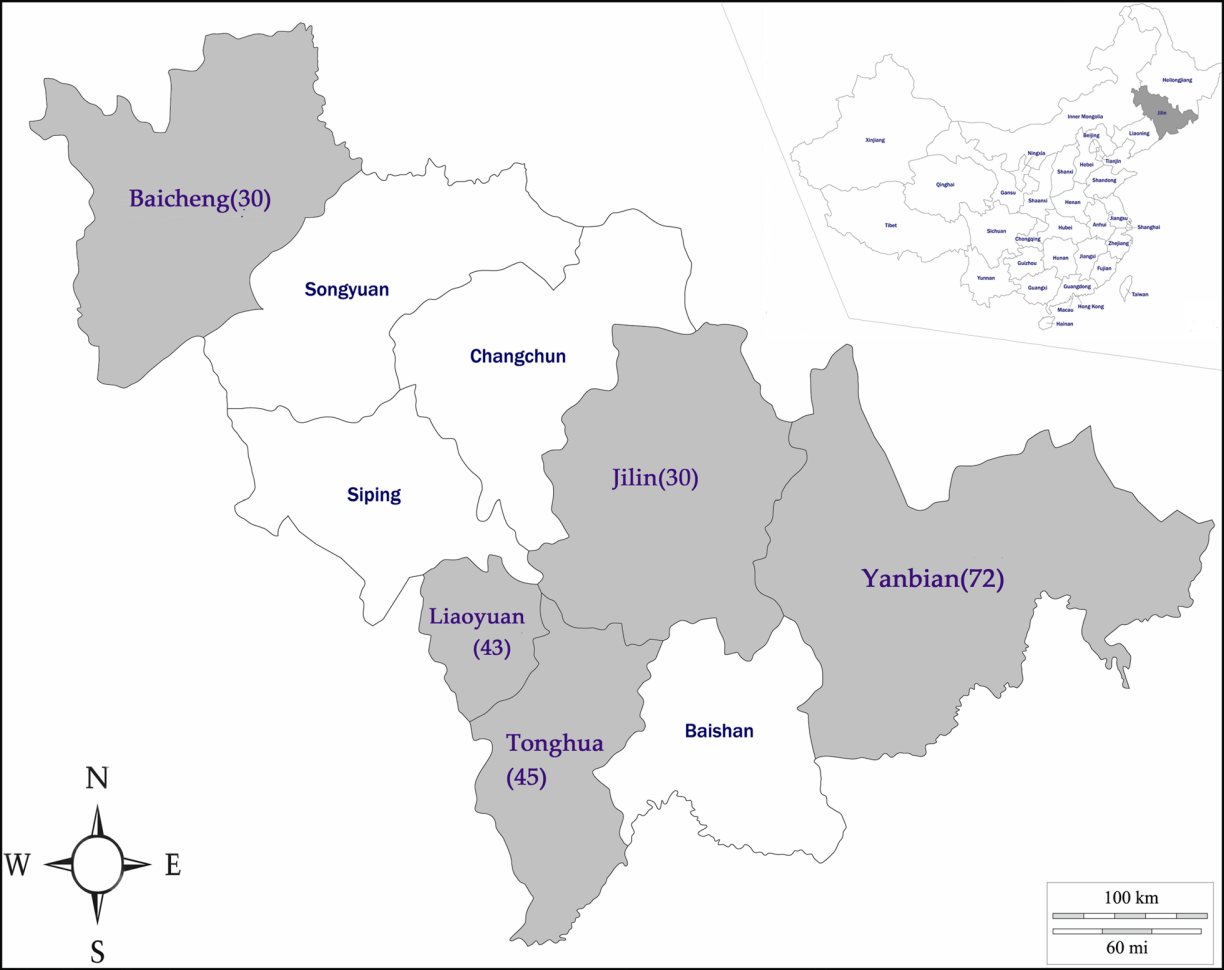
Map of the sampling districts in Jilin, China. The number within each region represents the sample size collected in this study

From March 2018 to October 2019, a total of 220 blood samples of equids were collected from the following five counties in Jilin Province (Fig. 1): Liaoyuan; Baicheng; Jilin; Yanbian; and Tonghua. Blood was aseptically collected from the jugular vein from each animal using K2-EDTA vacuum tubes (Solarbio, Beijing, China) and carefully stored at -20 °C until DNA extraction. A predesigned epidemiological questionnaire consisting of information regarding the region, sex, management, and host species of the animals was filled out with the help of farm owners to evaluate the risk factors associated with prevalence of equine piroplasm.

### DNA extraction and PCR amplification

The genomic DNA was extracted from 200 μl of each equine blood sample using a blood extraction kit (Vazyme, Nanjing, China) according to the manufacturer’s recommendations, and was subsequently stored at -20 °C until further use. The extracted DNA was used as a template for conventional PCR and nested PCR (nPCR) amplification based on previously published procedures using *T. equi*-specific primers to target the *18S* rRNA gene [14] and the *B. caballi*-specific BC48 gene [15]. The PCR reaction was conducted in a 25 μl reaction mixture comprised of 1 μl of each primer (10 pmol), 3 μl template DNA (45–55 ng/μl), 2 μl dNTP mix (TaKaRa, Dalian, China), 2.5 μl of 10× Ex Taq buffer, 0.25 μl Ex Taq (TaKaRa), and 15.25 μl distilled water. Genomic DNA extracted from the blood of horses naturally infected with *T. equi* and *B. caballi* was used as a positive control. Distilled water was used as a negative control. The nucleotide sequences of the primers and procedures of the PCR cycling conditions are presented in Table 2.

**Table 2:** Primer sequences for the amplification of *T. equi* and/or *B. caballi*

Organism-s	Target gene	Assay	Product size (bp)	Primer	Sequence 5′–3′	Annealing temperature (°C)	Reference
*T. equi*	18S rRNA	PCR	435	P1	TCGAAGACGATCAGATACCGTCG	60	[14]
				P2	TGCCTTAAACTTCCTTGCGAT		
*B. caballi*	BC48	Nested PCR	454	P3	ACGAATTCCCACAACAGCCGTGTT	55	[15]
				P4	ACGAATTCGTAAAGCGTGGCCATG		
				P5	GGGCGACGTGACTAAGACCTTATT		
				P6	GTTCTCAATGTCAGTAGCATCCGC		
*T. equi*/*B. caballi*	18S rRNA	Nested PCR	~ 1400	P7	GAAAYTGCGAATGGCTCATTAM	57	[13]
				P8	CACCGGATCACTCGATCGGTAGG		
				P9	GGATAACCGTGSTAATTSTAGGGC		
				P10	GTGTGTACAAAGGGCAGGGACG		

### Sequencing and phylogenetic analysis

Amplicons from positive PCR products were purified using a Gel Extraction Kit (OMEGA, Norcross, GA, USA) and were then cloned into a PMD 18T-Simple Vector (TaKaRa). The recombined clone plasmids were transformed into competent DH5α cells (TaKaRa). Following transformation, the precisely identified amplicons were sequenced by Shanghai Yingweijieji Biotechnology Company.

**Fig. 2:**
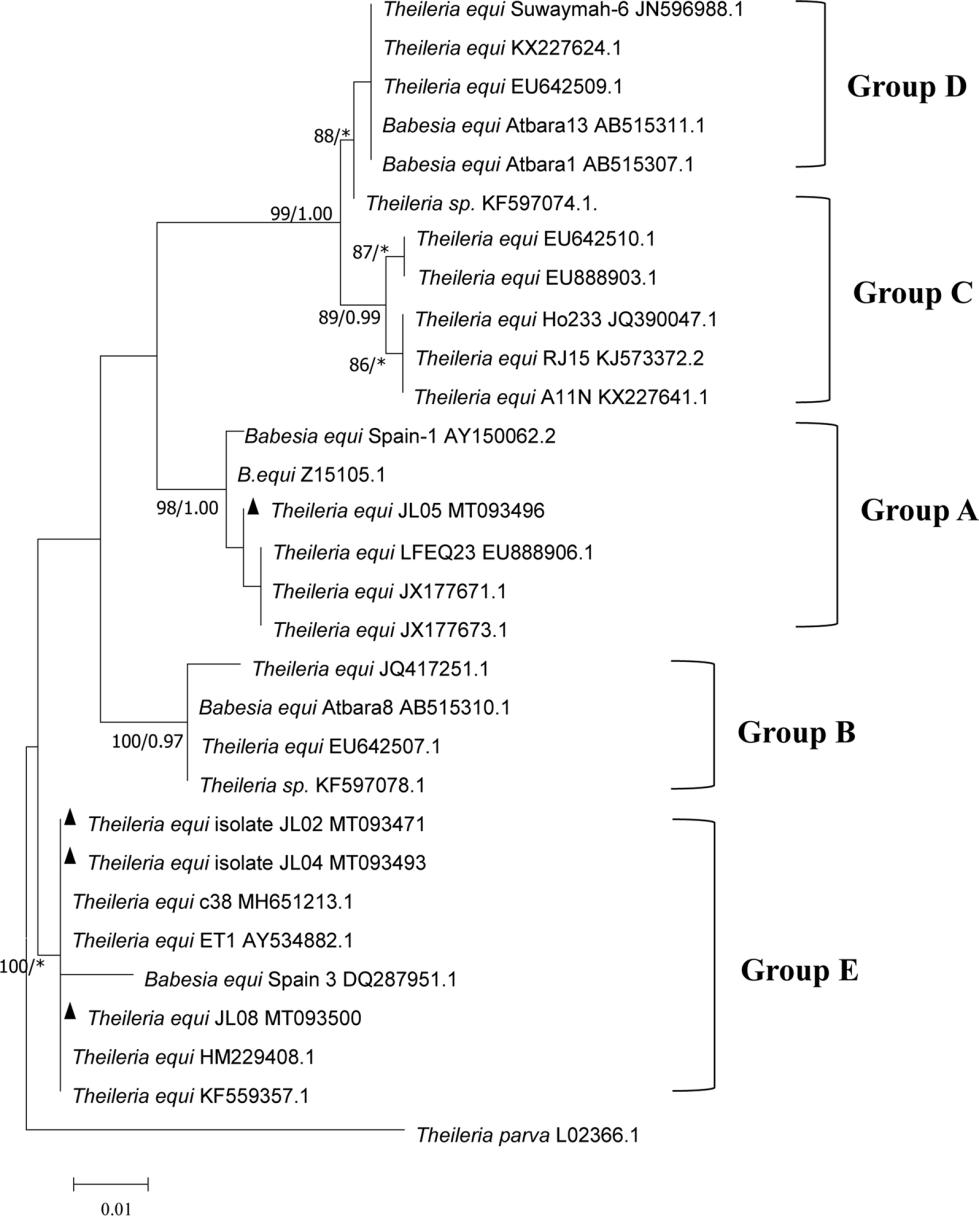
Phylogenetic tree based on the partial sequences of the *18S* rRNA gene of *T. equi*. The ML tree and Bayesian inference were implemented by MEGA7 with a Tamura 3-parameter model and MrBayes3.2 with the GTR + G + I model, respectively. The numbers at the nodes represent the ML bootstrap and BI posterior probability values. Bootstrap values < 70 and posterior probabilities < 0.95 are not shown. The gene sequences of *T. equi* obtained in the present study are indicated by a triangle. The five groups (A, B, C, D and E) represent the different *T. equi* genotypes

The obtained sequences were initially analyzed using the BLASTn program on the NCBI website. All sequences obtained in this study were aligned using ClustalW and then manually edited using Bioedit v.7.0.9 software (Fig. 2) [16]. Phylogenetic trees were constructed using the maximum likelihood (ML) (MEGA v.7.0) and Bayesian (MrBayes 3.2) methods [17]. For the ML analysis, a Tamura-3-parameter model and bootstrapping of 1000 replicates were selected to calculate the evolutionary relationship using MEGA v.7.0 software. The Bayesian analysis was performed using MrBayes 3.2 software. We ran four Monte Carlo Markov chains for 10^6^ generations, sampling every 10^3^ generations with the GTR + G + I model. The initial 25% samples were discarded as ‘burn-in’.

### Statistical analysis

The epidemiological data generated in this study were analyzed to identify which variables were associated with the equine piroplasm infection. A Fisher’s exact or chi-square test was performed to assess the significant differences among the regions, management, sex, and species based on the prevalence of *T. equi* and/or *B. caballi*. Variables yielding a significant association (*P*-value ≤ 0.05, two-tailed) were further applied in a stepwise backward logistic regression analysis [18]. Variance inflation factor (VIF) was calculated to test and avoid multicollinearity among variables. The fitness of the final models was assessed using a Hosmer-Lemeshow test, Negelkerke *R*^2^ test, and the observed *versus* predicted values (residual statistics) [19]. The 95% confidence intervals (95% CI), odds ratios (OR), and *P*-values were also computed. Differences were considered to be statistically significant if *P* < 0.05. All analyses were performed using SPSS software version 19.0 (IBM SPSS Statistics for Windows, Version 19.0. Armonk, IBM Corp., NY, USA) [20].

## Results

### Molecular detection of equine piroplasm

The PCR amplification results showed that the *T. equi* PCR amplified fragments of 435 bp (P1 and P2) from 61 samples had a positive rate of 22.7% (61/220). All of the surveyed samples were negative for *B. caballi* (P3–P6). Among the five regions studied, the highest prevalence of *T. equi* was recorded in Baicheng (43.3%, 95% CI: 24.5–62.2%), whereas the lowest prevalence was observed in Liaoyuan (20.9%, 95% CI: 8.3–33.6%). The prevalence among these two regions differed significantly (*P* < 0.05). The infection rates were detected in the other three regions of Tonghua (28.9%, 95% CI: 15.1–42.7%), Yanbian (26.4%, 95% CI: 16.0–36.8%), and Jilin (23.3%, 95% CI: 7.3–39.4%) (Table 3). The molecular prevalence of *T. equi* was also analyzed in the different categories of equids based on management, sex and species. The equids from the organized farms had a significantly lower positive rate (18.3%) compared to those from the unorganized farms (34.6%) (*P* < 0.05). The prevalence between the different sexes showed that the female equid population (32.1%) was more prone to infection compared with the males (16.1%) (*P* < 0.05). In this study, 192 horses and 28 donkeys/mules were compared for the prevalence of *T. equi*. The former had a higher prevalence (28.1%, 95% CI: 21.7–34.5%) than the latter group (25%, 95% CI: 7.9–42.1%) (Table 3).

**Table 3:** Results of the univariate analyses for the prevalence of *T. equi*

Variable	Samples tested (positive/total samples tested)	Prevalence (%)	95% CI	OR	95% CI	*P*-value
Region						
Liaoyuan	9/43	20.9	8.3–33.6	Ref		
Baicheng	13/30	43.3	24.5–62.2	2.89	1.03–8.09	0.04^*^
Jilin	7/30	23.3	7.3–39.4	1.15	0.38–3.53	0.80
Yanbian	19/72	26.4	16.0–36.8	1.35	0.55–3.34	0.51
Tonghua	13/45	28.9	15.1–42.7	1.54	0.58–4.08	0.39
Management						
Unorganized	44/127	34.6	26.3–43.0	Ref		
Organized	17/93	18.3	10.3–26.3	0.42	0.22–0.80	0.007^**^
Sex						
Male	10/62	16.1	6.7–25.5	Ref		
Female	51/158	32.3	24.9–39.6	2.48	1.17–5.27	0.016^*^
Species						
Horse	54/192	28.1	21.7–34.5	Ref		
Donkey/mule	7/28	25.0	7.9–42.1	0.85	0.34–2.12	0.73
Total	61/220	27.7	21.8–33.7			

*Abbreviations*: Ref, reference; 95% CI, confidence interval

*P < 0.05, **P < 0.01

The results of VIF analysis (VIF < 1.2) showed that there was no multicollinearity among all variables. In the first step of the risk factor analysis, four variables (region, farm management, equine species and sex) were assessed using a bivariate analysis. The results of the analysis in the univariate model indicated that two variables (management and sex) were sufficiently significant (*P *< 0.05) for further analysis. The region was also collected as a potential risk factor to be included in the multivariable analysis. A Hosmer-Lemeshow test (*χ*^2^ = 6.18, *df* = 4, *P *= 0.186) and Nagelkerke *R*^2^ (0.120) values suggested that this model was a good fit. The results of the multivariate logistic analysis suggested that females, equines of Baicheng, or unorganized farms were more likely to test positive for *T. equi* (Table 4).

**Table 4:** Results of the multivariate logistic analysis of risk factors for predicting *T. equi* infection in Jilin, China

Variable	Category	*P-*value	Odds ratio	95% CI
Region	All other regions	a		
	Baicheng	0.006	3.452	1.414–8.424
Management	Unorganized	a		
	Organized	0.020	0.456	0.235–0.886
Sex	Male	a		
	Female	0.012	2.924	1.269–6.737

*Abbreviations*: a, baseline; 95% CI, confidence interval

### Sequencing analysis

A total of 61 *T. equi 18S* rRNA gene sequences (approximately 1400 bp) were successfully obtained from the previous identified positive samples by nPCR, which further confirmed the results (435 bp) obtained from the PCR diagnosis. The BLAST analysis revealed four different haplotypes of the *18S* rRNA gene sequences in this study. Therefore, four *18S* rRNA sequences (MT093471, MT093493, MT093496 and MT093500) were deposited in the GenBank database. The new *T. equi 18S* rRNA gene sequences obtained in this study showed 96.8–99.9% sequence identity to each another. All new isolates, except for JL05 (GenBank: MT093496), which displayed the highest sequence identity (99.2%) with Chile (GenBank: MT463613) and had the highest sequence identities with the strains isolated from South Korea (GenBank: HM229407), varying from 99.8–100%.

### Phylogenetic analysis

The *T. equi* phylogenetic tree was constructed using the *18S* rRNA gene sequences obtained in our study and those previously published in GenBank. The tree topologies generated with the ML and BI analyses were generally similar. Therefore, the topology of the ML tree was presented with the support values (ML/BI) indicated on the branches. Phylogenetic analyses based on the *T. equi 18S* rRNA gene sequences in this study showed evidence of five main groups, which represent five genotypes, termed A, B, C, D and E [21] (Fig. 2). The results of the phylogenetic analysis indicated that the *T. equi 18S* rRNA gene sequences from Jilin Province obtained in the present study belonged to genotypes A and E. The new sequences of *T. equi* genotype E (MT093471, MT093493 and MT093500) obtained in this study shared 99.2–99.5% identities with a reference sequence from Spain (GenBank: DQ287951). Within group A, the *T. equi 18S* rRNA gene (GenBank: MT093496) sequence formed one clade with South Africa (GenBank: EU888906) and shared a 99.2% nucleotide identity. In addition, the two *T. equi* genotype groups, C and D, had a closer kinship, which was supported by the results of the statistical analysis (99% ML/1.00 BI).

## Discussion

The GenBank database of the National Center for Biotechnology Information (NCBI), contains published sequences of endemic *T. equi* and *B. caballi* isolates from more than 35 countries. The prevalence of EP occurs most frequently in the spring and autumn and is closely related to tick vector activity. Jilin Province is located within the middle of northeast China, which is rich in forest resources and animal species. This superior ecological environment provides a greater benefit to humans and is also a breeding ground for ticks. In this study, we found that the overall molecular prevalence of *T. equi* was 27.7%, which is significantly lower than the previous results (43.3%) detected by Xiong et al. [22]. This high degree of variability in the prevalence rates should be interpreted with extreme caution, due to differences in the sampling areas, sample size, animal species, and control programmes [23, 24]. However, infection with *B. caballi* was not identified in the present study, which could be attributed to the absence of competent tick vectors in the sampling areas, time and range. The existence of competent tick vectors is critical for maintaining the life-cycles of these parasites. There were 15 and 14 separate species of ticks reported as competent vectors for the transmission of *B. caballi* and *T. equi*, respectively [1]. However, the tick material collected from equines was absent and further studies must be implemented to confirm the competent tick vector responsible for the spread of EP in Jilin. Furthermore, equids infected with *B. caballi* are typically capable of naturally eliminating the infection, and imidocarb treatment has a reliably good curative effect at the recommended dosage for these parasites [25]. In contrast, once infected with *T. equi*, equids remain carriers throughout their lifetime and represent important reservoirs of this protozoan for naïve tick vectors [26, 27]. Due to the limitations associated with the sample size and narrow age range, most of the samples collected in this study were aged four to eight years of age. Therefore, there remains a need to establish the prevalence of *B. caballi* in Jilin.

In this study, we evaluated the risk factors considered to be associated with *T. equi* infection. It was observed that the samples collected from Baicheng had the highest positive rate (43.3%) compared to other areas. Baicheng is located in the northwest region of Jilin Province, which neighbors the grassland of Inner Mongolia and is characterized by abundant vegetation resources that provides a suitable environment for tick reproduction. This may be a crucial factor for greater infection of *T. equi* among equids in Baicheng; however, no significant differences were found among all other regions. The prevalence of *T. equi* infection in the organized farms (18.3%) was significantly lower than that detected in the unorganized farms (34.6%). Animals fed in an organized farm may have good management practices (e.g. deworming, vaccination, and veterinary care), which can effectively reduce the incidence of *T. equi* infection. However, animals within an unorganized farm are consistently fed by grazing or with other domestic animals, which may increase the risk of *T. equi* infection [28, 29]. The positive rate was significantly higher (32.3%) in the female population compared with the males (16.1%), which is consistent with the findings of other studies [18, 30]. This discrepancy in the prevalence of different sexes can be explained by the fact that the majority of the males sampled from farms in this study were stallions, which have greater value for breeding. In general, equines destined for reproductive activity (e.g. stallions) receive management considered to be ideal for this type of breeding *via* appropriate zootechnical buildings. Under such conditions, the animals receive balanced meals, systematic veterinary care, and sanitary control; thus, they are at a decreased risk of infestation with a tick vector and other reservoir hosts [31]. The relationship between sex hormones and susceptibility of males and females to protozoan infections has been studied in multiple species [32]. However, whether similar effects exist for equine piroplasm infection may require further research. The prevalence of *T. equi* infection was higher (28.1%) in the horse population as compared with donkeys/mules (25.0%), which differs from previous studies conducted in India and Italy [33, 34]. In these previous studies, the donkeys/mules possessed higher positive rates than the horses, because donkeys/mules are primarily used as working equids and are generally reared in poor living conditions, which may increase the chances of contact with infected tick vectors and other ruminants. This discrepancy in the prevalence within different regions may be attributed to differences in the purpose of husbandry. In this study, donkeys/mules are primarily raised for the purpose of food sources and leisure activities, which require stricter livelihood conditions and parasitic control programmes. Horses used for exhibition and sports typically have a higher prevalence of *T. equi* infection [31], and similar results were observed in this study.

Based on the analysis of the *18S* rRNA gene variants, we found two *T. equi 18S* rRNA genotypes, which were designated as genotypes A and E. Within group E, *T. equi* genotype E has recently been reported in Gansu (GenBank: MH651213), which shares 99.9–100% similarity with the isolates (MT093471, MT093493 and MT093500) obtained in our study [13]. The pairwise comparison indicated that there were no more than two nucleotide differences in both *Theileria* sequences, which confirmed that *T. equi* genotype E presented in these two areas should be considered a variation of the same strain. The severe pathogenicity associated with *T. equi* genotype E may be closely related to the clinical death of horses, which has been reported in both Spain and Greece [35, 36]. The results of our study indicate that the *T. equi* genotype E is widespread in Jilin Province. Although most of the cases in this study appeared to be asymptomatic, the prevalence of this genotype requires further research. Furthermore, one sequence belonging to *T. equi* genotype A that was first reported in South Africa (GenBank: Z15105) was identified in our study and has seldom been reported in China. Further information of the *T. equi* genotype A is needed to evaluate the association between this genotype with the prevalence of EP.

The occurrence of equine piroplasmosis has been reported in several regions of China, which has caused considerable economic loss to the equine industry. To understand the prevalence of EP over time, specimens were collected from five counties in Jilin Province to investigate the infection rate and analyze the epidemiology of EP. The findings of our study provided scientific evidence regarding the prevalence and geographical distribution of EP in Jilin, China, which is beneficial for controlling the source of infection, cut-off transmission routes, and to further protect susceptible equids.

## Conclusions

To our knowledge, this is the first study to investigate the genetic diversity of *T. equi* in equids from Jilin Province, China. The results of the present study revealed that the region was endemic for equine piroplasmosis with *T. equi* enzootic stability. Only the risk factors of sex and farm management were associated with *T. equi* infection in equids of the northeastern region in China. These factors should be used for the development or improvement of programmes for the prevention and control of equine piroplasmosis in the studied region.

## Data Availability

All data supporting the conclusions of this article are included within the article. The newly generated sequences were deposited in the GenBank database under the accession numbers MT093471, MT093493, MT093496 and MT093500.

## Abbreviations

EP: equine piroplasmosis
PCR: polymerase chain reaction
nPCR: nested PCR
*18S* rRNA: 18 Svedberg ribosomal ribonucleic acid
